# Assessment of Integrated Disease Surveillance Data Uptake in Community Health Systems within Nairobi County, Kenya

**DOI:** 10.24248/eahrj.v4i2.644

**Published:** 2020-11-26

**Authors:** Athanasio Japheth Omondi, Otieno George Ochieng, Khayo Eliud, Alison Yoos, Muli Rafael Kavilo

**Affiliations:** a Department of Health Management and Informatics, School of Public Health, Kenyatta University; b Improving Public Health Management for Action (IMPACT); c Ministry of Health Kenya; d Training Programs in Epidemiology and Public Health Interventions Network (TEPHINET) (Consultant); e Nairobi City County, Department of Integrated Disease Surveillance Nairobi City; f School of Economics, University of Nairobi

## Abstract

**Background:**

Kenya has since independence struggled to restructure its health system to provide services to its entire population especially in outbreak responses. The last decade has seen the country witness disease outbreaks across the country i.e. Rift Valley fever in June 2018, and Chikungunya and Dengue fever in Mombasa in February 2018. This exposed the country's lack of preparedness in handling outbreaks at grass root level. Outbreak incidences tend to prevail at community level before a public health action is established, with the situation becoming dire in the lower tier health facilities.

**Objective:**

The purpose of the study was to assess the uptake of Integrated Disease Surveillance Response (IDSR) health data and utilisation at community level health systems in the six sub counties within Nairobi County of Kenya.

**Methodology:**

The study used cross-sectional descriptive research design on a target population of 1840 community health workers. The study used Yamane formula to calculate the sample size of 371 respondents, selected using stratified sampling and simple random sampling methods. The logistic regression model was used to assess the benefits of Integrated Data Surveillance and Response data in health facilities across Nairobi County. Data was collected using questionnaires, analysis done using Statistical Packages for Social Sciences, and findings presented in form of tables and bar graphs.

**Results:**

The study had 315 questionnaires were duly filled and returned, representing 85% response rate. The findings showed that 268(85%) Healthcare Workers lacked training on using disease surveillance data; 236(75%) cited lack of tools for disease surveillance in facilities, while 173(55%)cited lack of timely IDSR data as hindrance to IDSR data uptake. The regression findings showed that training of healthcare workers on IDSR, installation of disease surveillance system tools, and timely collection and dissemination of surveillance data increases the likelihood of IDSR data uptake in community health facilities.

**Conclusion:**

The study concluded that IDSR system tools should be installed in community health facilities across the six sub counties in Nairobi County. Training should be emphasised to ensure all health care workers have the required skills to use the IDSR data. There is need to ensure IDSR data is collected and disseminated on time to make it available for interpretation and use by health care workers in their respective facilities.

## BACKGROUND

Public health surveillance is a continuous collection, analysis, and interpretation of health data systematically for purposes of planning, decision making, implementation, and evaluation of public health activities^1–3^. The Alma Ata Declaration of 1978 emphasises community involvement in health services as the essential components of the Primary Health Care (PHC) towards the pursuit “Health for All’ and “Community participation”, with many Sub Saharan Africa countries embracing this notion^4,5^. Integrated Disease Surveillance and Response (IDSR) is a unit of the healthcare that makes surveillance and laboratory data more usable in improving detection and prevention of illnesses and disease outbreaks, hence the need for exhaustive data gathering, thorough analysis, and proper dissemination of the information for effective decision making^6–8^. In Kenya, Community Based-disease Surveillance (CBS) remains active via Community Health Volunteers (CHVs), who detect and are the main reporters on cases that might otherwise not be reported to health care facilities at primary level for immediate action response^9,10^. They in turn integrate health events with health centres (tier I, II & III) for response. According to the Government of Kenya (GoK) Health Sector Strategic Plan, the healthcare tiers include: **Tier I**, also known as Community Health Services (comprises all community based activities, mainly health promotion, disease prevention, and identification of cases that require reporting to higher levels of care); **Tier II**, also known as Primary Care Level (comprises of maternity homes, dispensaries, and health centres); **Tier III** which comprises of county referral hospitals that are normally staffed by a particular county within Kenya; and **Tier IV** which encompasses all national referral hospitals i.e. Kenyatta National Hospital, Mathari Hospital, Moi Teaching and Referral Hospital, and the National Spinal Injury Referral Hospital^6,11^.

Despite this progress made in the implementation of IDSR (Integrated disease surveillance & response), challenges still exist^12^. Cholera Outbreak in the month of July 2017 affected 6 Sub-Counties in Nairobi namely Kamukunji, Langata, Dagoretti, Embakasi, Starehe and Ruaraka. The outbreak had 64 confirmed cases, 317 probable cases, with 4 deaths, Case Fatality Rate (the proportion of deaths within a designated population of “cases” over the course of the disease) which is 1%^13^. However, it is noted that the cases were preventable if early response had been initiated.^14^

### Problem Gap

Kenya has made good progress in IDSR implementation with focal persons in most sub counties and electronic reporting at county level. Health facilities are the primary sources of disease data, even though their reporting rates have been below the target of 80% reporting rate.^9,15^ Nairobi County residents can access health facilities within a radius of 7 Km. The doctor-population ratio in Nairobi County is 1:7,143 while the nurse-population ratio is 1:887^16^. Despite this, outbreaks and emergencies still exist and response as a result of decision making is wanting, hence there was the need to assess the utilisation of routine data for decision making in health facilities in Kenya^10,13,14^. Despite the progress made in the implementation of IDSR, data analysis by the health care system remains sub-optimal, and thus, events-based incidences prevail at community without established public health action to avert the event^4,8,17^. With Kenya's adoption of decentralised system of government, there is greater call for community empowerment and involvement in health system and decision making. The study therefore sought to assess the uptake of Integrated Disease Surveillance and Response (IDSR) data in community health systems within Nairobi County.

## METHODOLOGY

### Research Design

The study used cross-sectional descriptive research design. The Office for Human Research Protection (OHRP) defines a descriptive study as one in which information is collected without changing the environment, and is conducted to demonstrate relationships between things^18^. A descriptive study can involve a one-time interaction with groups of items also known as cross sectional study or a study that might follow individuals over time, also known as longitudinal study^19^.

### Sample Size Calculation

The target population of the study was 1,840 community health workers which comprised of nurses, clinicians, public health officers, medical officers, community health assistants, lab technicians, and pharmacists in health centres and dispensaries within Nairobi County. The study used Yamane^20^. Formula used to calculate the sample size of 371 respondents (health care workers)^21^.

**Figure:**
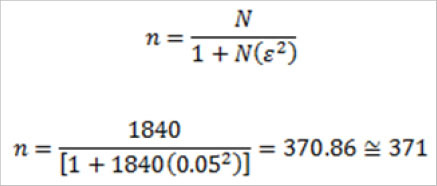


### Sampling and Data Collection

Nairobi County has 58 public health facilities spread across 6 sub counties. During sampling, each sub county was divided into 5 strata namely nurses, clinicians, medical officers, public health officers, and Community Health Volunteers (CHVs). Each stratum was then subjected to simple random sampling, with a total of 371 health care workers being selected from the entire target population. Data was therefore collected from 58 public health facilities in the 6 sub counties in Nairobi, with each sub county being a DSR resource centre. Data collection was carried out by 6 Research Assistants (RA), with each RA being allocated a DSR resource centre (Sub County) to handle. The study mainly utilised English language during data collection, with RAs utilising Swahili National language to elaborate on points that respondents found difficult to comprehend in English.

**FIGURE 1.**
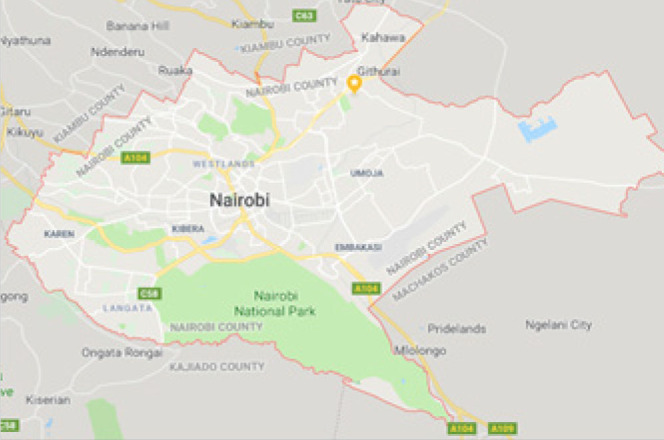
Study areas-Map of Nairobi City

### Data Analysis

In the pre-test of the research instrument, validity and reliability of the questionnaire was assessed, with the reliability outcome showing Cronbach Alpha of 0.72, implying that the instrument was suitable to serve the intended purpose^21^. Data obtained was analysed thematically using Statistical Package for Social Sciences (IM SPSS, Chicago - United States of America) software version 23, with socio-demographic descriptive analysis, frequency tables, as well as logistic regression analysis being carried on the relationship between training of HCWs, availability of IDSR system tools, timely dissemination of IDSR data (independent variables), and uptake of IDSR data in community health facilities (dependent variable). The logistic regression model was used to assess the likelihood of dependent variables influencing uptake of IDSR data in health facilities across Nairobi County. The Logistic regression model used was as follows:

**Figure:**
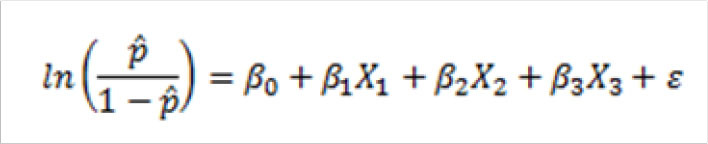


Where

– Training of HCWs

– Availability of IDSR sytem tools

– Timely dissemination of IDSR data

– Explanatory coefficients for i=1,2,3

The study adopted the 95% confidence level in regression of the model, with only p-values less than 0.05 (p<.05) being used in the findings of the logistic model. This is because the p-values of less than 0.05 clearly indicate that the explanatory variables used in the model have a statistically significant influence on the dependent variable^22,23^. It is on this premise that the study established that all the 3 factors i.e. Community health workers’ training on IDSR data, IDSR system tools, and availability of monthly IDSR data, are key factors in uptake of IDSR data in health facilities, since all the 3 variables were of p-values less than 0.05 (p<.05), indicating that the variables were statistically significant to explain variations in the model. The study findings were presented in form of tables and bar graphs.

### Ethical Considerations

Ethical approval and clearance was sought and obtained from the National Commission for Science, Technology, and Innovation (NACOSTI), with the ethical review process approving the study to proceed under certificate number NACOSTI/P/18/53954/26335. In addition, the study sought for permission from the Nairobi County Government, the Ministry of Health, and various health facilities to allow the study to be carried out. Verbal and written consent was obtained from all participants before interviews were conducted, with all questionnaires being assigned numbers to ensure anonymity of the data collected.

## RESULTS

### Descriptive results for health care workers component

371 questionnaires were distributed to the respondents, with a significant return rate of 315 (85%) questionnaires being recorded. Out of the 315 respondents taking part in the study, 186(59%) were male while the remaining 129(41%) were female. As shown in Table 1, the findings revealed that 69(21.91%) respondents had secondary school education, while 198(62.86%) had a college diploma education, with 30(9.52%) being university degree holders, while the remaining 18(5.71%) had a postgraduate degree. It was also noted that 68(21.59%) respondents were aged between 20-29 years, 114(36.19%) were aged between 30-39 years, 72(22.86%) were aged between 40-49 years, while the remaining 61(19.37%) were above 50 years.

**TABLE 1:** Socio-demographic characteristics

		Frequency	Percent
**Gender**	Male	186	59
	Female	129	41
	**Total**	**315**	**100**
**Age**	20 – 29 yrs	68	21.59
	30 – 39 yrs	114	36.19
	40 – 49 yrs	72	22.86
	50 yrs & above	61	19.37
	**Total**	**315**	**100**
**Education**	**Certifcate**	**69**	**21.91**
	Diploma	198	62.86
	Bachelor-	30	9.52
	Degree		
	Postgraduate	18	5.71
	Degree		
	**Total**	**315**	**100**

### Integrated Disease Surveillance and Response data Up-take:

According to the findings in Table 2, it was observed that 163(51.75%) respondents were not satisfied with the level of training offered, indicating that more training programs should be set up to improve health workers’ skills on IDSR data utilisation in their service delivery. 99(33.02%) respondents on the other hand were of contrary opinion that the CHW straining was enough to help them in IDSR data utilisation. However, 48(15.23%) respondents expressed reservation on the adequacy of training on IDSR data utilisation, citing lack of enough exposure and exchange programmes on IDSR data. The responses for training on IDSR data uptake shows a mean of 3.46475 and standard deviation of 0.95654, which implies that majority of the respondents agree with the assertion that is necessary to train health workers on utilisation of IDSR data in health facilities within Nairobi County.

**TABLE 2:** Uptake of IDSR data

Variable		Frequency	Percentage
Strongly Disagree		79	25.08
Disagree		84	26.67
Not sure		48	15.23
Agree		51	16.19
Strongly Agree		53	16.83
Total		315	100
**Variable**	**N**	**Mean**	**Std-Deviation**
CHW Training-on IDSR	315	2.37563	0.95654
IDSR System-tools	315	3.46475	1.00342
Availability of-IDSR monthly-data	315	2.98481	0.94926

## DISCUSSION

The study assessed the IDSR data uptake by health care workers at the community level in public health facilities within Nairobi County. It is argued that routine health care data generated by health care providers play a major role in facilitating integration between individual health and public health interventions after analysis^24–26^. The demographic factors considered included gender, age, and education of HCWs. The study findings further showed that more than 66% of the respondents had challenges with understanding IDSR data due to lack of analytical skills, while 34% reported to having the requisite technical skills to understand and utilise IDSR data. The CDC emphasises training to enhance the knowledge and skills of healthcare workers so that they may effectively use the data obtained from the surveillance system to improve patient and healthcare personnel safety^2,8,9^. It is therefore imperative that the HCWs in community health facilities within Nairobi County be trained on IDSR data analysis and utilisation.

### Factors influencing IDSR data Uptake

Regression of the logistic model gave the association between independent variables (factors of IDSR) and the uptake of Integrated Disease Surveillance and Response data as shown in the regression model:

**Figure:**
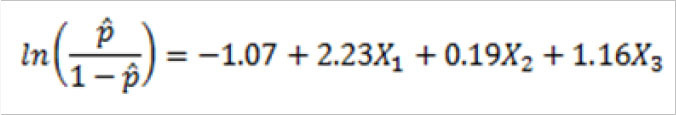


According to the logistic regression odds ratio in Table 3, training CHWs implies that healthcare workers are 4 times more likely to use IDSR data, while availability of IDSR system tools in health facilities, and timely collection and dissemination of IDSR data increase the likelihood of IDSR data uptake by 3 and 5 times respectively. The study findings concur with those carried out in Malawa^2^ and Uganda^25^ that developing information technology infrastructure in health facilities and ensuring timely dissemination of disease surveillance data will necessitate achievement of IDSR goals in countries within Sub Saharan Africa. It is therefore evident that availability of IDSR tools makes it possible for health facilities to generate and disseminate data, which is key in transformation of preparedness of developing countries in dealing with disease outbreaks^2,3,27^.

**TABLE 3:** Factors affecting IDSR data uptake

	Logistic Mode Coeffcient	Chi-Square	P-Value	Odds-Ratio
(Constant)	-1.066			
CHW Training on IDSR	2.233^**^	2.132	0.033	4.10
Availability of IDSR System Tools	0.187^*^	1.042	0.013	3.25
Timely Disse-mination of IDSR Data	1.158^**^	1.098	0.016	4.91

### Strengths and Limitations

The study reveals challenges facing uptake of IDSR data in community level health facilities within Nairobi County. This will make it easier for facility management and the Ministry of Health to put necessary measures and improve disease outbreak preparedness.

The study however had various limitations, key among them being the choice of the study to include only government-sponsored health facilities at community level, which left out private-owned and faith-based health facilities that are quite a considerable number in the Kenyan capital city. Further studies should therefore consider carrying out similar studies in all health facilities within Nairobi City County, including public, private, and faith-based health facilities.

## CONCLUSION

Following the study findings, it can be concluded that training of CHWs is key to the uptakes of IDSR data. CHWs are street-level bureaucrats in any healthcare system, and if well trained, they ensure civic education in their daily interactions with their patients, thereby ensuring successful implementation of government policies^5,15,24^. Installation of disease surveillance systems in health facilities enable the management to detect and curtail any disease outbreak in its early stages, thereby making it possible to avert disease outbreaks and epidemics^4,7,14,28^. There is therefore need to train all community health care workers on how to interpret and use IDSR data, as well as installing disease surveillance systems in health facilities to increase uptake of IDSR data.

## RECOMMENDATIONS

Following the study findings, it is recommended that:

- More emphasis should be put on training to ensure all health care workers have the required skills to use the IDSR data.
- There is need to ensure IDSR data is disseminated on time (in this case monthly) to make it available for interpretation and use by health care workers in their respective facilities. Health facilities should be fittedwith ICT infrastructure to enable installation of IDSR system tools in all health facilities within Nairobi County.
